# Generalizability of VICTORION-1 PREVENT enrollment criteria to the United States population

**DOI:** 10.1016/j.ajpc.2025.100957

**Published:** 2025-03-08

**Authors:** Rahul Aggarwal, Deepak L. Bhatt, Marc P. Bonaca, Catrin Deck, Anastasia Lesogor, Manesh R. Patel, Erik S.G. Stroes, Pam R. Taub, Stephan Windecker

**Affiliations:** a Brigham and Women's Hospital Heart and Vascular Center, Harvard Medical School, Boston, MA, USA; b Mount Sinai Fuster Heart Hospital, Icahn School of Medicine at Mount Sinai, New York, NY, USA; c Division of Cardiology, Department of Medicine, University of Colorado School of Medicine, Aurora, CO, USA; d Novartis Pharma AG, Basel, Switzerland; e Duke Clinical Research Institute and Division of Cardiology, Duke University, Durham, NC, USA; f Department of Vascular Medicine, Amsterdam University Medical Center, Amsterdam, Netherlands; g Department of Cardiovascular Medicine, University of California San Diego School of Medicine, La Jolla, CA, USA; h Department of Cardiology, Bern University Hospital, Inselspital, University of Bern, Freiburgstrasse, CH, 3010 Bern, Switzerland

**Keywords:** Cardiovascular risk, PCSK9 inhibition, Primary prevention

## Abstract

**Background:**

VICTORION-1 PREVENT (V-1P) is an ongoing trial evaluating inclisiran for lipid lowering in patients with high cardiovascular (CV) risk without established atherosclerotic CV disease (ASCVD). This study evaluates the generalizability of V-1P enrollment criteria to the US population and their clinical comorbidity and CV risk factor burden.

**Methods:**

Data from National Health and Nutrition Examination Surveys (2015-March 2020) were used to determine nationally representative estimates. Inclusion criteria were low-density lipoprotein cholesterol (LDL-C) of 70–189 mg/dL and a 10-year ASCVD risk of ≥20% or 7.5%-19.9% with two CV risk enhancers. The pooled cohort equations (PCE) was used to stratify ASCVD risk in primary analysis. Estimates of the US population were compared with the V-1P eligible population.

**Results:**

The V-1P eligible population included 23,837,940 adults. Compared with US adults ages 40-79 years, V-1P eligible adults had higher mean 10-year ASCVD risk by PCE (21.1% [95% CI: 20.1%-22.2%] vs 10.0% [95% CI: 9.4%-10.6%]). The V-1P eligible population also had higher rates of hypertension (85.4% [95% CI: 81.6%-89.1%] vs 59.4% [95% CI: 56.7%-62.2%], diabetes (35.6% [95% CI: 31.3%-40.0%] vs 18.7% [95% CI: 16.9%- 20.5%]) and metabolic syndrome (81.6% [95% CI: 78.4%-84.7%] vs 51.1% [48.3%- 53.9%]). Adults meeting V-1P eligibility had high levels of LDL-C (117.8 mg/dL [95% CI: 114.3 mg/dL-121.2 mg/dL]) and low statin use (36.7% [95% CI: 31.9%-41.5%]).

**Conclusions:**

Many primary prevention patients have high CV risk, significant comorbidity burden, and are eligible for lipid-lowering therapy, yet rates of treatment are low. Public health interventions to improve CV risk factor management are necessary.

## 1 Introduction

VICTORION-1 PREVENT (V-1P) is an ongoing randomized clinical trial evaluating the efficacy and safety of inclisiran, a siRNA lipid lowering therapy targeting proprotein convertase subtilisin/kexin type 9 (PCSK9) gene expression, in patients with high cardiovascular (CV) risk but without established atherosclerotic CV disease (ASCVD) [1].

In this study, we evaluate the generalizability of V-1P inclusion criteria to the US population and assess the clinical comorbidity and CV risk factor burden of the target population.

## 2 Methods

Data from the National Health and Nutrition Examination Surveys were used (NHANES 2015-March 2020, N = 25,531) to determine national representative estimates [2]. Adults aged 40–79 years in the fasting subsample of NHANES were identified. Patients were required to have a low-density lipoprotein cholesterol (LDL-C) of 70–189 mg/dL and a 10-year ASCVD risk of ≥20% or 7.5%−19.9% with two CV risk enhancers. Risk enhancers included elevated high-sensitivity CRP (hsCRP, ≥2 mg/L), inflammatory conditions (psoriatic or rheumatoid arthritis), low estimated glomerular filtration rate (eGFR, <60), metabolic syndrome, or family history of a heart attack (defined in NHANES as a close relative with a heart attack before 50 years of age). LDL-C estimates were based on the Friedewald equation [3]. Exclusion criteria included ASCVD, liver disease, and pregnancy. ASCVD exclusion was based on self-report of coronary heart disease, angina, heart attack, or stroke. Liver disease was defined as alanine aminotransferase or aspartate aminotransferase ≥150 U/L or self-report of liver disease. Pregnancy was based on self-report.

In the primary analysis, the pooled cohort equations (PCE) were used for ASCVD risk stratification [4]. In sensitivity analyses the PREVENT ASCVD equations were used [5]. Estimates of the US population 40–79 years were compared with the V-1P eligible populations. Survey weights were used to project national estimates. NHANES is approved by the National Center for Health Statistics ethics review board.

## 3 Results

**Fig. 1:**
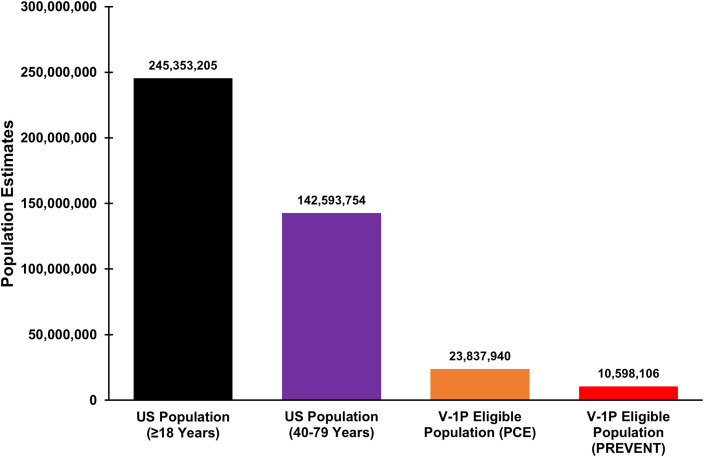
VICTORION-1 PREVENT Eligibility in the United States. Estimates of the US population meeting VICTORION-1 PREVENT (V-1P) eligibility. Four cohorts are shown: the US population ≥18 years of age, US population 40–79 years of age, V-1P eligible population using the pooled cohort equations (PCE), and the V-1P eligible population using the PREVENT equations. Estimates are nationally representative of the US Population using NHANES survey weights.

The projected US population ≥18 years consists of 245,353,205 adults, with 142,593,754 (58.1%) adults 40–79 years of age (Fig. 1). The V-1P eligible population included 23,837,940 (16.7%) adults, with mean age 65.7 years and 44.2% female. Compared with US adults 40–79 years, V-1P eligible adults had higher 10-year mean ASCVD risk by PCE (21.1% [95% CI: 20.1%−22.2%] vs 10.0% [95% CI: 9.4%−10.6%]).

The V-1P eligible population had higher rates of hypertension (85.4% [81.6%−89.1%] vs 59.4% [56.7%−62.2%]), diabetes (35.6% [31.3%−40.0%] vs 18.7% [16.9%−20.5%]), and metabolic syndrome (81.6% [78.4%−84.7%] vs 51.1% [48.3%−53.9%] than the US population 40–79 years. Both populations had high but similar levels of LDL-C (117.8 mg/dL [114.3–121.2 mg/dL] vs 115.3 mg/dL [113.0–117.6 mg/dL]) and low rates of statin use (36.7% [31.9%−41.5%] vs 28.4% [25.9%−30.8%]). When determining V-1P eligibility by PREVENT, 10,598,106 (7.4%) adults met eligibility criteria. This population had the highest 10-year mean ASCVD risk by PCE (25.3% [23.3%−27.3%]), with majority of patients having hypertension, diabetes, and metabolic syndrome (Table 1). Full characteristics among these populations are shown in the Table 1

**Table 1:** Characteristics of the US Population Compared with the VICTORION-1 PREVENT Eligible Population.

	**US population** **(40–79 years)**	**V-1P eligible population (PCE)**	**V-1P eligible population (PREVENT)**	**P-Value** **(US vs V-1P eligible PCE)**	**P-Value** **(US vs V-1P eligible PREVENT)**
**Population Estimate**	142,593,754 (131,871,266–153,316,242)	23,837,940 (21,143,795–26,532,085)	10,598,106 (8,796,009–12,400,203)		
**Mean Age, Years**	57.2 (56.6–57.9)	65.7 (65.0–66.4)	67.1 (65.8–68.4)	<0.001	<0.001
**Sex**				0.002	0.22
Female, %	47.9 (46.1–49.8)	44.2 (39.9–48.6)	47.3 (40.2–54.5)		
Male, %	52.1 (50.2–53.9)	55.8 (51.4–60.1)	52.7 (45.5–59.8)		
**Race/Ethnicity**				0.002	0.09
Non-Hispanic White, %	66.3 (62.5–70.2)	64.4 (58.2–70.6)	66.0 (58.5–73.6)		
Mexican American, %	6.8 (5.1–8.4)	6.4 (4.2–8.7)	5.4 (2.7–8.1)		
Other Hispanic, %	6.7 (5.2–8.2)	6.8 (4.7–9.0)	6.7 (3.6–9.7)		
Non-Hispanic Black, %	10.9 (8.6–13.3)	15.5 (11.5–19.5)	15.2 (10.4–19.9)		
Non-Hispanic Asian, %	5.6 (4.2–7.0)	3.7 (2.4–5.0)	2.8 (1.2–4.4)		
Other Race & Ethnicities, %	3.7 (2.8–4.7)	3.1 (1.6–4.6)	4.0 (0.9–7.0)		
**Ratio of Family Income to Poverty Level**				0.05	0.002
0–99, %	11.4 (9.5–13.3)	13.5 (10.0–16.9)	14.1 (9.8–18.5)		
100–199, %	16.8 (15.1–18.4)	19.1 (15.7–22.5)	21.3 (14.9–27.8)		
200–299, %	14.8 (13.0–16.7)	18.2 (13.1–23.2)	23.3 (15.3–31.3)		
≥300, %	57.0 (53.9–60.1)	49.2 (43.6–54.9)	41.2 (32.5–50.0)		
**Insured**, %	89.8 (87.8–91.9)	93.7 (91.3–96.1)	95.0 (91.9–98.1)	0.004	0.02
**Mean Body Mass Index, kg/m^2^**	30.0 (29.6–30.4)	31.7 (31.1–32.3)	32.8 (32.0–33.7)	<0.001	<0.001
Underweight, %	0.9 (0.5–1.2)	0.0 (0.0–0.1)	0.1 (0.0–0.3)		
Normal Weight, %	21.5 (19.1–23.9)	10.7 (7.6–13.7)	4.1 (1.8–6.4)		
Overweight, %	35.4 (33.4–37.4)	31.9 (27.6–36.2)	30.2 (23.2–37.2)		
Obese Weight, %	42.2 (39.6–44.7)	57.4 (53.4–61.4)	65.6 (59.4–71.8)		
**Hypertension (130/80), %**	59.4 (56.7–62.2)	85.4 (81.6–89.1)	91.3 (87.5–95.2)	<0.001	<0.001
**Mean Systolic Blood Pressure, mmHg**	125.3 (124.4–126.2)	134.9 (132.9–136.8)	135.8 (132.6–139.0)	<0.001	<0.001
<120, %	40.5 (37.9–43.0)	20.9 (16.0–25.9)	22.7 (15.2–30.2)		
120–139, %	40.9 (38.5–43.3)	40.9 (36.5–45.4)	33.8 (27.2–40.5)		
≥140, %	18.7 (16.7–20.6)	38.1 (32.6–43.7)	43.5 (35.5–51.5)		
**Mean Diastolic Blood Pressure, mmHg**	73.4 (72.8–74.0)	72.8 (71.4–74.3)	72.4 (70.1–74.7)	0.45	0.41
<80, %	72.4 (70.4–74.4)	72.5 (68.6–76.3)	74.0 (67.4–80.5)		
80–89, %	20.5 (18.7–22.3)	18.4 (14.7–22.0)	18.5 (12.8–24.2)		
≥90, %	7.1 (6.0–8.2)	9.2 (6.7–11.7)	7.5 (4.1–11.0)		
**Diabetes**, %	18.7 (16.9–20.5)	35.6 (31.3–40.0)	52.7 (46.0–59.4)	<0.001	<0.001
**Mean A1c,**	5.9 (5.8–5.9)	6.2 (6.2–6.3)	6.5 (6.3–6.7)	<0.001	<0.001
<6.5, %	86.1 (84.3–87.8)	74.5 (70.4–78.7)	61.0 (53.2–68.7)		
6.5–7.9, %	9.8 (8.3–11.3)	19.3 (15.2–23.4)	30.3 (23.0–37.7)		
≥8.0, %	4.1 (3.2–5.0)	6.2 (4.6–7.8)	8.7 (5.1–12.4)		
**Fasting Blood Glucose, mg/dL**	114.4 (112.8–115.9)	125.3 (122.0–128.6)	136.3 (129.5–143.2)	<0.001	<0.001
**Metabolic Syndrome**, %	51.1 (48.3–53.9)	81.6 (78.4–84.7)	94.7 (91.9–97.5)	<0.001	<0.001
**History of Smoking**, %	47.7 (44.6–50.9)	51.8 (46.7–57.0)	56.6 (50.0–63.1)	0.18	0.02
**Mean Total Cholesterol, mg/dL**	194.4 (191.6–197.2)	195.1 (190.7–199.4)	194.1 (188.0–200.2)	0.76	0.92
**Mean Low Density Lipoprotein Cholesterol, mg/dL**	115.3 (113.0–117.6)	117.8 (114.3–121.2)	116.7 (112.0–121.3)	0.20	0.54
0–99, %	34.4 (31.5–37.3)	31.5 (26.2–36.7)	33.1 (25.5–40.8)		
100–160, %	54.9 (52.2–57.5)	58.8 (54.5–63.1)	57.2 (49.1–65.3)		
160–189, %	8.2 (6.9–9.5)	9.7 (6.6–12.8)	9.7 (4.7–14.6)		
≥190, %	2.5 (1.7–3.3)	0.0 (0.0–0.0)	0.0 (0.0–0.0)		
**Mean High Density Lipoprotein Cholesterol, mg/dL**	55.5 (54.5–56.5)	50.3 (48.8–51.7)	48.8 (46.5–51.0)	<0.001	<0.001
**Mean Triglycerides, mg/dL**	119.4 (115.1–123.6)	135.3 (126.9–143.6)	143.5 (129.7–157.3)	<0.001	0.001
**Statin Use**, %	28.4 (25.9–30.8)	36.7 (31.9–41.5)	38.1 (30.3–46.0)	<0.001	0.009
**Estimated Glomerular Filtration Rate, ml/min/1.73m^2^**	90.4 (89.4–91.3)	81.2 (79.0–83.3)	75.5 (71.6–79.4)	<0.001	<0.001
**Median High Sensitivity C-Reactive Protein, mg/L**	1.9 (1.8–2.1)	3.3 (2.8–3.6)	3.7 (3.3–4.3)	0.002	0.001
**Mean 10 Year PCE**, %	10.0 (9.4–10.6)	21.1 (20.1–22.2)	25.3 (23.3–27.3)	<0.001	<0.001
**Mean 10 Year PREVENT**, %	4.9 (4.7–5.2)	9.5 (9.1–9.9)	11.7 (11.1–12.2)	<0.001	<0.001

Nationally representative estimates of the US population in the National Health and Nutrition Examination Survey (2015-March 2020). V-1P eligible population consists of those meeting criteria for VICTORION-1 PREVENT (V-1P), with 10-year atherosclerotic cardiovascular disease risk determined by either pooled cohort equations (PCE) or PREVENT equations. Age, sex, race, and ethnicity were based on self-report. P-values were comparison of the US population age 40–79 years that was eligible and was not eligible for V-1P. T-tests were used for continuous variables and chi squared test for categorical variables.

## 4 Discussion

When applying V-1P eligibility criteria to the US population, over 23 million adults meet criteria for inclusion. Many of these patients had high CV risk or significant burden of risk factors.

While prior evidence suggests low use of lipid lowering therapy and low rates of intensification in secondary prevention populations [[6], [7], [8]], lipid lowering therapy use in high-risk primary prevention populations are not well described. This study adds to prior data, suggesting low rates of therapy use in high-risk primary prevention populations. Guidelines recommend lipid lowering therapy for high-risk patients with 10-year ASCVD risk ≥7.5% [9]. V-1P eligible patients were at very high 10-year ASCVD risk (mean >20%), but despite this elevated risk, statin use was low (<37%). Even though there have been population level improvements in lipid levels [10,11], the findings of this study indicate that mean LDL-C levels in the US remain high among high CV risk patients. When applying V-1P criteria by PREVENT, fewer patients met V-1P eligibility, but still over 10 million were eligible. These patients were of the highest CV risk based on risk estimation.

Patients eligible for V-1P, irrespective of risk stratification method, had low rates of hypertension, dyslipidemia, and hyperglycemia control, indicating a need for urgent strategies to improve CV risk factor management in these patients. Limitation includes underestimation of population size and CV risk due to inability to identify coronary calcifications or other risk enhancing factors such as inflammatory bowel disease or early menopause in NHANES. Data for peripheral artery disease, carotid artery disease, or electrocardiographic data to identify ASCVD were not available.

In the US, many primary prevention patients have high CV risk and are eligible for lipid lowering therapy, yet rates of therapy use are low. V-1P eligibility applies to many patients, who are at high CV risk and need further interventions to improve CV risk factor management.

## 5 Data sharing

National Health and Nutrition Examination Survey data is publicly available from the National Center for Health Statistics (https://www.cdc.gov/nchs/nhanes/index.html)

## Author agreement

All authors have seen and approved the final version of the manuscript being submitted. The article is the authors' original work, hasn't received prior publication and isn't under consideration for publication elsewhere.

The author roles were as follows:

Conceptualization: RA, DLB

Formal analysis: RA

Investigation: All authors

Methodology: All authors

Supervision: DLB

Writing – original draft: RA

Writing – review and editing: All authors

## Funding

Dr. Aggarwal receives research training support from the National Heart, Lung, and Blood Institute grant 5T32HL007604.

## CRediT authorship contribution statement

**Rahul Aggarwal:** Writing – review & editing, Writing – original draft, Supervision, Investigation, Formal analysis, Conceptualization. **Deepak L. Bhatt:** Writing – review & editing, Supervision, Methodology, Investigation, Conceptualization. **Marc P. Bonaca:** Writing – review & editing, Methodology, Investigation. **Catrin Deck:** Writing – review & editing, Methodology, Investigation. **Anastasia Lesogor:** Writing – review & editing, Methodology, Investigation. **Manesh R. Patel:** Writing – review & editing, Methodology, Investigation. **Erik S.G. Stroes:** Writing – review & editing, Methodology, Investigation. **Pam R. Taub:** Writing – review & editing, Methodology, Investigation. **Stephan Windecker:** Writing – review & editing, Methodology, Investigation.

## Declaration of competing interest

Dr. Aggarwal is involved in research funded by the Bristol Myers Squibb-Pfizer alliance, Novartis, Amarin, Cleerly, and Lexicon. He has previously served as a consultant for Lexicon. Dr. Bhatt discloses the following relationships - Advisory Board: Angiowave, Bayer, Boehringer Ingelheim, CellProthera, Cereno Scientific, E-Star Biotech, High Enroll, Janssen, Level Ex, McKinsey, Medscape Cardiology, Merck, NirvaMed, Novo Nordisk, Stasys; Tourmaline Bio; Board of Directors: American Heart Association New York City, Angiowave (stock options), Bristol Myers Squibb (stock), DRS.LINQ (stock options), High Enroll (stock); Consultant: Broadview Ventures, Corcept Therapeutics, GlaxoSmithKline, Hims, SFJ, Summa Therapeutics, Youngene; Data Monitoring Committees: Acesion Pharma, Assistance Publique-Hôpitaux de Paris, Baim Institute for Clinical Research (formerly Harvard Clinical Research Institute, for the PORTICO trial, funded by St. Jude Medical, now Abbott), Boston Scientific (Chair, PEITHO trial), Cleveland Clinic, Contego Medical (Chair, PERFORMANCE 2), Duke Clinical Research Institute, Mayo Clinic, Mount Sinai School of Medicine (for the ENVISAGE trial, funded by Daiichi Sankyo; for the ABILITY-DM trial, funded by Concept Medical; for ALLAY-HF, funded by Alleviant Medical), Novartis, Population Health Research Institute; Rutgers University (for the NIH-funded MINT Trial); Honoraria: American College of Cardiology (Senior Associate Editor, Clinical Trials and News, ACC.org; Chair, ACC Accreditation Oversight Committee), Arnold and Porter law firm (work related to Sanofi/Bristol-Myers Squibb clopidogrel litigation), Baim Institute for Clinical Research (formerly Harvard Clinical Research Institute; AEGIS-II executive committee funded by CSL Behring), Belvoir Publications (Editor in Chief, Harvard Heart Letter), Canadian Medical and Surgical Knowledge Translation Research Group (clinical trial steering committees), CSL Behring (AHA lecture), Cowen and Company, Duke Clinical Research Institute (clinical trial steering committees, including for the PRONOUNCE trial, funded by Ferring Pharmaceuticals), HMP Global (Editor in Chief, Journal of Invasive Cardiology), Journal of the American College of Cardiology (Guest Editor; Associate Editor), Level Ex, Medtelligence/ReachMD (CME steering committees), MJH Life Sciences, Oakstone CME (Course Director, Comprehensive Review of Interventional Cardiology), Piper Sandler, Population Health Research Institute (for the COMPASS operations committee, publications committee, steering committee, and USA national co-leader, funded by Bayer), WebMD (CME steering committees), Wiley (steering committee); Other: Clinical Cardiology (Deputy Editor); Patent: Sotagliflozin (named on a patent for sotagliflozin assigned to Brigham and Women's Hospital who assigned to Lexicon; neither I nor Brigham and Women's Hospital receive any income from this patent); Research Funding: Abbott, Acesion Pharma, Afimmune, Aker Biomarine, Alnylam, Amarin, Amgen, AstraZeneca, Bayer, Beren, Boehringer Ingelheim, Boston Scientific, Bristol-Myers Squibb, Cardax, CellProthera, Cereno Scientific, Chiesi, CinCor, Cleerly, CSL Behring, Faraday Pharmaceuticals, Ferring Pharmaceuticals, Fractyl, Garmin, HLS Therapeutics, Idorsia, Ironwood, Ischemix, Janssen, Javelin, Lexicon, Lilly, Medtronic, Merck, Moderna, MyoKardia, NirvaMed, Novartis, Novo Nordisk, Otsuka, Owkin, Pfizer, PhaseBio, PLx Pharma, Recardio, Regeneron, Reid Hoffman Foundation, Roche, Sanofi, Stasys, Synaptic, The Medicines Company, Youngene, 89Bio; Royalties: Elsevier (Editor, Braunwald's Heart Disease); Site Co-Investigator: Cleerly. Dr. Bonaca is the Executive Director of CPC, a non-profit academic research organization affiliated with the University of Colorado, that receives or has received research grant/consulting funding between August 2021 and present from: Abbott Laboratories, Agios Pharmaceuticals, Inc., Alexion Pharma, Alnylam Pharmaceuticals, Inc., Amgen Inc., Angionetics, Inc., Anthos Therapeutics, Array BioPharma, Inc., AstraZeneca and Affiliates, Atentiv LLC, Audentes Therapeutics, Inc., Bayer and Affiliates, Bristol-Meyers Squibb Company, Cambrian Biopharma, Inc., Cardiol Therapeutics Inc., CellResearch Corp., Cleerly Inc., Cook Regentec LLC, CSL Behring LLC, Eidos Therapeutics, Inc., EP Trading Co. Ltd., Epizon Pharma, Inc., Esperion Therapeutics, Inc., Everly Well, Inc., Exicon Consulting Pvt. Ltd., Faraday Pharmaceuticals, Inc., Foresee Pharmaceuticals Co. Ltd., Fortress Biotech, Inc., HDL Therapeutics Inc., HeartFlow Inc., Hummingbird Bioscience, Insmed Inc., Ionis Pharmaceuticals, Janssen and Affiliates, Kowa Research Institute, Inc., Lexicon Pharmaceuticals, Inc., Medimmune Ltd., Merck & Affiliates, Nectero Medical Inc., Novartis Pharmaceuticals Corp., Novo Nordisk, Inc., Osiris Therapeutics Inc., Pfizer Inc., PhaseBio Pharmaceuticals, Inc., Prairie Education and Research Cooperative, Prothena Biosciences Limited, Regeneron Pharmaceuticals, Inc., Regio Biosciences, Inc., Sanofi-Aventis Groupe, Silence Therapeutics PLC, Smith & Nephew plc, Stealth BioTherapeutics Inc., VarmX, Virta Health Corporation. Dr. Deck is an employee of Novartis Pharma AG, Switzerland. Dr. Lesogor is an employee of Novartis Pharma AG, Switzerland.

Dr. Stroes has received adboard/consultancy fees paid to institution by Amgen, Novartis, Novo-Nordisk, Merck, Ionis, Astra-Zeneca. Dr. Windecker reports research, travel or educational grants to the institution without impact on his personal remuneration from Abbott, Abiomed, Amgen, Astra Zeneca, Bayer, Bbraun, Biotronik, Boehringer Ingelheim, Boston Scientific, Bristol Myers Squibb, Cardinal Health, CardioValve, Cleerly Inc., Cordis Medical, Corflow Therapeutics, CSL Behring, Daiichi Sankyo, Edwards Lifesciences, Farapulse Inc. Fumedica, GE Medical Systems, Gebro Pharma, Guerbet, Idorsia, Inari Medical, InfraRedx, Janssen-Cilag, Johnson & Johnson, Medalliance, Medicure, Medtronic, Merck Sharp & Dohm, Miracor Medical, Neucomed, Novartis, Novo Nordisk, Organon, OrPha Suisse, Pharming Tech, Pfizer, Philips AG, Polares, Regeneron, Sanofi-Aventis, Servier, Siemens Healthcare, Sinomed, SMT Sahajanand Medical Technologies, Terumo, Vifor, V-Wave, Zoll Medical. Dr. Windecker serves as advisory board member and/or member of the steering/executive group of trials funded by Abbott, Abiomed, Amgen, Astra Zeneca, Bayer, Boston Scientific, Biotronik, Bristol Myers Squibb, Edwards Lifesciences, MedAlliance, Medtronic, Novartis, Polares, Recardio, Sinomed, Terumo, and V-Wave with payments to the institution but no personal payments. He is also a member of the steering/executive committee group of several investigator-initiated trials that receive funding by industry without impact on his personal remuneration. Dr. Taub reports a relationship with Novartis, Esperion Therapeutics Inc., Amarin, Amgen Inc., Novo Nordisk Inc., Medtronic Inc., Edwards, Inc., Boehringer Ingelheim, Jazz Pharmaceuticals, Milestone Pharmaceuticals, Bayer, and Lilly that includes consulting or advisory role. Dr. Taub serves as an ad advisory board member and/or member of the steering/executive committee of trials funded by Novartis, Merck, Amgen, Arrowhead, Clearly, Medtronic, CSL Behring and Lilly. Dr. Patel reports research grants with Novartis, Bayer, Jansen, Idorsia, NHBLI. Dr. Patel has advisory/consulting relationships with Esperion, Bayer, Jansen, and Idorsia.
